# Computed Tomography-Based Comparison of Anterior and Posterior Cortical Thickness Around the Interfragmentary Screw Fixation Level in Weber B Lateral Malleolar Fractures †

**DOI:** 10.3390/jcm15145610

**Published:** 2026-07-17

**Authors:** Jaehyung Lee, Sungwoo Cho, Jae Yong Park

**Affiliations:** 1 Department of Orthopedic Surgery, Seoul Now Hospital, Anyang-si 14058, Gyeonggi-do, Republic of Korea; vical719@naver.com; 2 Department of Orthopedic Surgery, Hallym University Sacred Heart Hospital, Hallym University College of Medicine, Anyang-si 14068, Gyeonggi-do, Republic of Korea; antoniocho94@gmail.com

**Keywords:** ankle fracture, lateral malleolar fracture, distal fibula, cortical thickness, interfragmentary screw, lag screw, computed tomography

## Abstract

**Background/Objectives:** Cortical morphology around the screw fixation level may influence screw purchase in Weber B lateral malleolar fractures. This study compared anterior and posterior cortical thickness of the distal fibula around the interfragmentary screw fixation level using preoperative computed tomography (CT) images matched with postoperative radiographs. **Methods:** This retrospective radiographic study included 155 patients who underwent operative treatment for Weber B lateral malleolar fractures using an interfragmentary screw between 2019 and 2023. Patients with Weber A or C fractures, no preoperative CT, fixation without an interfragmentary screw, previous ipsilateral lateral malleolar fracture or surgery, or unreliable CT measurement were excluded. The levels at which the screw crossed the anterior and posterior cortices were identified on postoperative radiographs and transferred to sagittal CT images. **Results:** The anterior cortex was significantly thicker than the posterior cortex (2.44 ± 0.82 mm vs. 1.39 ± 0.66 mm; mean difference, 1.05 mm; 95% confidence interval, 0.94–1.17; *p* < 0.001). This anterior cortical predominance was observed across all sex- and age-based subgroups. Men had greater anterior and mean cortical hickness than women, while patients aged <60 years had greater cortical thickness than those aged ≥60 years. **Conclusions:** Around the interfragmentary screw fixation level in Weber B lateral malleolar fractures, the anterior cortex was consistently thicker than the posterior cortex. Given the limitations of radiograph-to-CT matching, the results should be interpreted as demonstrating a relative regional pattern rather than exact absolute cortical thickness values. This pattern may provide useful reference information when planning screw fixation.

## 1. Introduction

Ankle fractures are among the most common injuries treated in orthopedic practice, with reported annual incidences of approximately 120–170 fractures per 100,000 persons [1,2,3]. Lateral malleolar fractures represent the most common subtype, accounting for approximately 55% of ankle fractures in a large population-based study [1]. In studies using the Danis–Weber classification, type B fractures have been reported to account for approximately 42% of adult ankle fractures [4]. In Weber B lateral malleolar fractures, operative treatment is commonly indicated when displacement or instability is present, and stable fixation of the distal fibula is essential for restoring fibular length, rotation, and ankle mortise congruence [5,6].

For simple oblique Weber B fractures, interfragmentary lag screw fixation combined with neutralization plating has traditionally been used to obtain compression across the fibular fracture site [6]. However, fixation strategies for lateral malleolar fractures continue to vary. Several studies have examined whether an interfragmentary lag screw is necessary when plate fixation is used [7,8,9], whereas others have investigated alternative screw trajectories, including posterior-to-anterior lag screw placement [10,11,12]. Alternative fixation constructs for Weber B fractures have also been evaluated biomechanically [13]. These variations suggest that local bone morphology around the screw fixation level may be relevant when planning fixation, particularly because cortical thickness can influence screw purchase and fixation mechanics.

Plain radiographs remain the standard initial imaging modality for acute ankle trauma and suspected ankle fractures [14]. However, in fractures selected for operative fixation, CT is commonly used as an adjunct to delineate fracture morphology, associated posterior malleolar or Chaput fragments, syndesmotic involvement, comminution, and other features relevant to operative planning. Beyond fracture-line assessment, CT also enables evaluation of local osseous morphology that cannot be readily assessed on plain radiographs alone.

Recent imaging-based studies have further emphasized the importance of fracture-specific and region-specific assessment of the distal fibula. Three-dimensional computed tomography (CT) studies and fracture-mapping analyses have described the morphology and distribution of type B lateral malleolar fractures [15,16], while CT-based morphometric studies have evaluated distal fibular anatomy, medullary canal dimensions, and potential corridors for intramedullary fixation [17,18]. In addition, high-resolution peripheral quantitative CT studies have shown that distal fibular microarchitecture varies according to subregion, age, and sex [19]. These studies provide valuable anatomical information for implant design, intramedullary fixation, and screw corridor planning.

However, most previous morphometric studies evaluated intact fibulae, healthy volunteers, or cadaveric specimens, and measurements were generally performed at predefined distances from the lateral malleolar tip or along fixed anatomical levels [17,18,19]. Even fracture-mapping and three-dimensional morphology studies have primarily focused on fracture line distribution and fragment morphology rather than the anterior and posterior cortical thickness encountered around the level of interfragmentary screw fixation [15,16]. Therefore, available data may not fully reflect the local cortical morphology encountered during operative fixation of real Weber B fracture patients.

This distinction is clinically important. In surgically treated Weber B fractures, the interfragmentary screw is inserted according to the fracture configuration and the surgeon’s reduction strategy, and screw purchase is obtained at the level where the screw crosses the posterior and anterior cortices. Therefore, morphometric data around the actual screw fixation level may provide more directly relevant information for fixation planning than measurements obtained from intact fibulae or fixed anatomical levels.

The purpose of this study was to compare posterior and anterior cortical thickness of the distal fibula around the interfragmentary screw fixation level in surgically treated Weber B lateral malleolar fractures using preoperative CT images matched with postoperative radiographs. We hypothesized that the anterior cortex would be thicker than the posterior cortex around the screw fixation level and that this anterior cortical predominance would be maintained across sex- and age-based subgroups.

## 2. Materials and Methods

### 2.1. Study Design and Patients

This retrospective radiographic study included consecutive patients who underwent surgical treatment for ankle fractures at our institution between January 2019 and December 2023. The study was approved by the Institutional Review Board of Hallym University Sacred Heart Hospital (IRB No. 2024-10-004), and the requirement for informed consent was waived because of the retrospective design.

Patients were eligible if they underwent operative treatment for an acute Weber B lateral malleolar fracture using an interfragmentary screw with a neutralization plate and had preoperative computed tomography (CT) scans available for analysis. During the study period, 382 patients underwent surgical treatment for ankle fractures at our institution. Exclusion criteria were applied sequentially in the order listed below, and each patient was counted only once according to the first applicable exclusion criterion. Patients were excluded for the following reasons: age 16 years or younger (n = 21); absence of preoperative CT imaging (n = 8); ankle fractures without lateral malleolar involvement, such as isolated medial malleolar fractures or combined medial and posterior malleolar fractures (n = 78); Weber type A or C lateral malleolar fractures (n = 52); fixation without an interfragmentary screw (n = 18); previous ipsilateral ankle surgery or fracture (n = 8); and unreliable cortical thickness measurement because of severe comminution, marked displacement, poor image quality, or other technical reasons (n = 42). After applying these criteria, 155 patients were included in the final analysis. At our institution, standard ankle radiographs were obtained as the initial imaging study for patients with suspected ankle fractures. For fractures that were minor, stable, or considered likely to be managed non-operatively, evaluation was generally based on standard ankle radiographs without additional CT. When operative fixation was planned, preoperative CT was generally obtained to evaluate fracture morphology, associated posterior malleolar or Chaput fragments, syndesmotic involvement, comminution, and other features relevant to surgical planning, except in urgent situations or when CT could not be performed because of patient-related factors.

Patient age, sex, side of injury, and diagnosis were reviewed. Body mass index was not analyzed because height and weight data were not consistently available in this retrospective radiographic dataset. Based on the diagnostic description, fracture patterns were categorized as isolated lateral malleolar fractures, bimalleolar fractures or bimalleolar-equivalent injuries, and trimalleolar fractures or trimalleolar-equivalent injuries.

### 2.2. Radiographic Measurements

Anterior and posterior cortical thicknesses of the distal fibula were measured around the level of interfragmentary screw fixation. In this study, the screw fixation level was operationally defined as the level at which the interfragmentary screw crossed the posterior and anterior cortices of the fibula on postoperative radiographs.

First, on postoperative ankle radiographs, a horizontal reference line was drawn through the tip of the lateral malleolus. The vertical distances from this reference line to the points where the interfragmentary screw intersected the posterior and anterior cortices of the fibula were measured. Using a cross-reference tool on preoperative CT images, the reference level corresponding to the lateral malleolar tip was identified on reformatted images. The same distances measured on postoperative radiographs were then transferred to the sagittal CT image to estimate the corresponding screw-cortex intersection levels.

**Figure 1:**
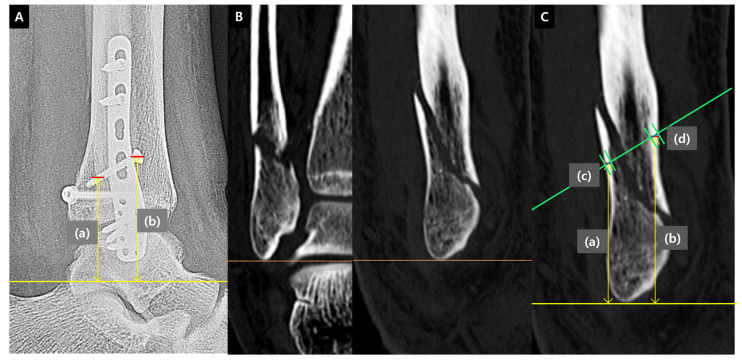
Measurement technique for anterior and posterior cortical thickness around the interfragmentary screw fixation level. (**A**) On postoperative ankle radiographs, a horizontal reference line was drawn through the tip of the lateral malleolus. The vertical distances from this reference line to the points where the interfragmentary screw crossed the posterior (a) and anterior (b) cortices of the fibula were measured. (**B**) On preoperative computed tomography (CT) images, the reference level corresponding to the lateral malleolar tip was identified using a cross-reference tool. (**C**) The distances measured on postoperative radiographs were transferred to the sagittal CT image to estimate the screw-cortex intersection levels. The posterior (c) and anterior (d) cortical thicknesses were then measured at these estimated levels.

At these estimated levels, posterior and anterior cortical thicknesses were measured on sagittal CT images. Cortical thickness was defined as the shortest distance across the cortical bone at each measurement point. All CT scans were acquired with a slice thickness of 1 mm. Measurements were performed using the institutional picture archiving and communication system. The overall measurement process, including identification of the screw-cortex intersection levels on postoperative radiographs and transfer of these levels to preoperative CT images, is illustrated in Figure 1.

To assess measurement reliability, 50 cases were randomly selected from the study cohort. For interobserver reliability, two observers (S.C. and J.L.) independently measured anterior and posterior cortical thickness. For intraobserver reliability, one observer (S.C.) repeated the measurements after an interval of 2 weeks, blinded to the initial measurements. Interobserver and intraobserver reliability were evaluated using intraclass correlation coefficients (ICCs) with 95% confidence intervals.

### 2.3. Statistical Analysis

Continuous variables are presented as means with standard deviations, and categorical variables are presented as numbers and percentages. Mean cortical thickness was calculated as the average of anterior and posterior cortical thickness. The primary analysis was the within-patient comparison between anterior and posterior cortical thickness around the interfragmentary screw fixation level. Paired *t*-tests were used for this comparison, and the mean difference with 95% confidence interval was calculated.

Patients were stratified by sex and by age using a predefined cutoff of 60 years. Between-group comparisons of anterior cortical thickness, posterior cortical thickness, and mean cortical thickness were performed using Welch’s *t*-test to account for potential inequality of variances between groups. Sex- and age-based subgroup analyses were considered exploratory. Additional exploratory subgroup analyses were performed according to fracture pattern categories. In these analyses, anterior and posterior cortical thicknesses were compared within each fracture pattern subgroup using paired *t*-tests.

Interobserver and intraobserver reliability were assessed using ICCs with 95% confidence intervals. ICCs were calculated using a two-way random-effects model for absolute agreement, single measures, according to published recommendations for ICC reporting [20]. ICC values were interpreted as poor (<0.50), moderate (0.50–0.75), good (0.75–0.90), or excellent (>0.90). Statistical analyses were performed using IBM SPSS Statistics version 27.0 (IBM Corp., Armonk, NY, USA). A *p*-value < 0.05 was considered statistically significant.

## 3. Results

A total of 155 patients were included in the final analysis. There were 76 men (49.0%) and 79 women (51.0%), and the mean age was 48.1 ± 17.3 years. The mean age was 43.6 ± 19.4 years in men and 52.5 ± 13.7 years in women. Overall, 102 patients (65.8%) were aged <60 years and 53 patients (34.2%) were aged ≥60 years. Among men, 55 patients were aged <60 years and 21 were aged ≥60 years; among women, 47 patients were aged <60 years and 32 were aged ≥60 years. The injured side was right in 72 patients and left in 83 patients. Based on diagnostic descriptions, the cohort included 39 isolated lateral malleolar fractures, 39 bimalleolar fractures or bimalleolar-equivalent injuries, and 77 trimalleolar fractures or trimalleolar-equivalent injuries (Table 1).

The mean anterior cortical thickness around the interfragmentary screw fixation level was 2.44 ± 0.82 mm, whereas the mean posterior cortical thickness was 1.39 ± 0.66 mm. The anterior cortex was significantly thicker than the posterior cortex, with a mean difference of 1.05 mm (95% confidence interval [CI], 0.94–1.17; *p* < 0.001). This anterior cortical predominance was observed in the overall cohort and across all sex- and age-based subgroups (Table 2).

In additional exploratory analyses according to fracture pattern, the anterior cortex remained significantly thicker than the posterior cortex in all fracture pattern subgroups. In isolated lateral malleolar fractures, anterior and posterior cortical thicknesses were 2.37 ± 0.70 mm and 1.27 ± 0.52 mm, respectively, with a mean difference of 1.10 mm (95% CI, 0.87–1.33; *p* < 0.001). In bimalleolar fractures or bimalleolar-equivalent injuries, the corresponding values were 2.48 ± 0.85 mm and 1.42 ± 0.57 mm, with a mean difference of 1.07 mm (95% CI, 0.83–1.30; *p* < 0.001). In trimalleolar fractures or trimalleolar-equivalent injuries, the corresponding values were 2.46 ± 0.87 mm and 1.43 ± 0.75 mm, with a mean difference of 1.02 mm (95% CI, 0.86–1.19; *p* < 0.001).

When patients were stratified by sex, men had significantly greater anterior cortical thickness than women (2.66 ± 0.90 mm vs. 2.24 ± 0.68 mm; *p* = 0.001). Posterior cortical thickness was numerically greater in men than in women, but this difference did not reach statistical significance (1.49 ± 0.76 mm vs. 1.30 ± 0.52 mm; *p* = 0.075). Mean cortical thickness was significantly greater in men than in women (2.07 ± 0.74 mm vs. 1.77 ± 0.51 mm; *p* = 0.004) (Table 3).

Using 60 years as the age cutoff, patients aged <60 years had greater cortical thickness than those aged ≥60 years in the overall cohort. Anterior cortical thickness was 2.54 ± 0.84 mm in patients aged <60 years and 2.26 ± 0.76 mm in those aged ≥60 years (*p* = 0.039). Posterior cortical thickness was 1.47 ± 0.70 mm and 1.24 ± 0.54 mm, respectively (*p* = 0.028), and mean cortical thickness was 2.00 ± 0.68 mm and 1.75 ± 0.54 mm, respectively (*p* = 0.014) (Table 3).

In sex-specific age subgroup analyses, younger men had numerically greater cortical thickness than older men, although the differences were not statistically significant. In men, anterior cortical thickness was 2.69 ± 0.93 mm in the younger group and 2.57 ± 0.83 mm in the older group (*p* = 0.581); posterior cortical thickness was 1.54 ± 0.78 mm and 1.36 ± 0.73 mm, respectively (*p* = 0.353); and mean cortical thickness was 2.11 ± 0.76 mm and 1.96 ± 0.69 mm, respectively (*p* = 0.412).

Among women, younger patients had significantly greater cortical thickness than older patients. Anterior cortical thickness was 2.36 ± 0.69 mm in younger women and 2.06 ± 0.64 mm in older women (*p* = 0.049). Posterior cortical thickness was 1.39 ± 0.59 mm and 1.17 ± 0.36 mm, respectively (*p* = 0.044), and mean cortical thickness was 1.87 ± 0.57 mm and 1.61 ± 0.37 mm, respectively (*p* = 0.015). These findings suggest that age-related differences in cortical thickness were more evident in women than in men.

Interobserver and intraobserver reliability were assessed in 50 randomly selected cases. The interobserver ICCs for anterior and posterior cortical thickness were 0.905 (95% CI, 0.845–0.944) and 0.885 (95% CI, 0.819–0.928), respectively. The intraobserver ICCs for anterior and posterior cortical thickness were 0.952 (95% CI, 0.922–0.970) and 0.932 (95% CI, 0.891–0.959), respectively. These values indicated good-to-excellent measurement reliability.

## 4. Discussion

The principal finding of this study was that, in surgically treated Weber B lateral malleolar fractures fixed with an interfragmentary screw, the anterior cortex of the distal fibula was consistently thicker than the posterior cortex around the screw fixation level. Although the absolute cortical thickness values should be interpreted cautiously because of the limitations of radiograph-to-CT matching, the anterior cortical predominance was marked and was observed in the overall cohort as well as across sex- and age-based subgroups.

The present study was designed to provide fracture-specific morphometric information at the level where interfragmentary screw fixation is actually performed. Recent CT-based studies and fracture-mapping analyses have highlighted the value of three-dimensional assessment in characterizing type B lateral malleolar fractures and identifying region-specific fracture patterns [15,16]. Other CT-based and high-resolution imaging studies have described distal fibular anatomy, medullary canal morphology, cortical indices, and subregional variation in bone microarchitecture [17,18,19]. These studies provide valuable anatomical information for implant design, intramedullary fixation, and screw corridor planning. However, many previous morphometric measurements were obtained from intact or non-fracture fibulae, cadaveric specimens, or fixed anatomical levels, often at predefined distances from the lateral malleolar tip [17,18,19]. In contrast, the present study used postoperative radiographs to identify the interfragmentary screw level and matched this level to preoperative CT images in patients with actual Weber B fractures. This approach may better reflect the cortical morphology encountered during operative fixation, although it inevitably includes some degree of measurement uncertainty.

The most important interpretation of our findings is not the exact numerical thickness of each cortex, but the consistent regional difference between the anterior and posterior cortices. The process of transferring the screw-cortex intersection level from postoperative radiographs to preoperative CT images may be affected by radiographic projection, limb rotation, screw obliquity, CT reconstruction, slice selection, and fracture displacement. Therefore, the reported values should not be regarded as precise anatomical measurements of cortical thickness. Nevertheless, the magnitude and consistency of the anterior–posterior difference suggest that the anterior cortex is generally thicker than the posterior cortex around the level where interfragmentary screw fixation is performed.

This regional pattern may have practical relevance for screw fixation in Weber B lateral malleolar fractures. Interfragmentary lag screw fixation with neutralization plating remains a commonly used technique, but the necessity of lag screw fixation and the optimal screw trajectory remain areas of ongoing discussion [7,8,9,10,11,12,13]. In lag screw fixation, the near cortex is overdrilled to create a glide hole, and compression is generated through purchase in the far fragment [12]. If the far cortical segment is very thin or fragile, screw purchase may be insufficient, which may reduce the effectiveness of interfragmentary compression. The present findings suggest that the posterior cortex is generally thinner than the anterior cortex around the screw fixation level. This information may therefore be considered when selecting screw trajectory, deciding whether a lag screw technique is appropriate, or choosing positional screw fixation instead of compression screw fixation, particularly in patients with thin cortices or potentially poor bone quality. However, because this study did not directly compare fixation methods or screw trajectories, these findings should be regarded as morphometric reference information rather than evidence favoring a specific fixation technique. Therefore, the approximately 1 mm anterior–posterior difference observed in this study should be interpreted as a statistically significant morphometric finding rather than direct evidence of biomechanical superiority or improved clinical outcomes.

From a surgical perspective, the relatively thin posterior cortex may have negative implications when the posterior cortex functions as the principal site of far-cortex purchase, such as during conventional anterior-to-posterior lag screw fixation. In such cases, a thin posterior cortex may theoretically increase the risk of inadequate screw purchase, cortical stripping, or limited interfragmentary compression, particularly in patients with poor bone quality. One possible implication of the present findings is that, when a lag screw technique is selected, a posterior-to-anterior screw trajectory may be considered in selected cases so that the relatively thicker anterior cortex serves as the far cortex for screw purchase [10,11,12]. However, the present study does not provide direct biomechanical or clinical evidence to recommend a posterior-to-anterior trajectory routinely, nor does it support routine use of smaller-diameter screws. A smaller-diameter screw may reduce local cortical demand, but it may also reduce screw purchase and compression; therefore, screw diameter should be selected according to fragment size, cortical quality, and standard fixation principles. Similarly, plate fixation without an interfragmentary screw has been reported to provide satisfactory outcomes in selected Weber B or distal fibular fractures [7,8,9]. However, the decision to omit interfragmentary fixation should not be based solely on the morphometric findings of the present study. Intraoperative assessment of screw purchase and construct stability remains essential. When posterior cortical purchase appears insufficient, surgeons may consider alternative strategies such as modifying the screw trajectory, including posterior-to-anterior insertion, using a positional screw rather than a lag screw, relying on stable neutralization plate fixation, or omitting the interfragmentary screw if adequate stability can be achieved with the plate alone.

The role of CT in Weber B lateral malleolar fractures should also be interpreted in a practical clinical context. Plain radiographs remain the standard initial imaging modality for ankle fractures [14]. CT can provide additional information regarding fracture morphology and surgical planning, but it also involves additional radiation exposure and cost compared with plain radiography [21]. In addition, CT protocols and indications may differ among institutions, and not every center routinely obtains CT for Weber B lateral malleolar fractures. Therefore, the findings of this study should not be interpreted as supporting CT solely for cortical thickness measurement. Rather, the results provide additional morphometric information that may be useful when CT is obtained as part of preoperative assessment for fractures selected for operative fixation.

Sex- and age-based analyses provided additional observations. Men had significantly greater anterior and mean cortical thickness than women, whereas posterior cortical thickness was numerically greater in men but did not reach statistical significance. Patients aged <60 years had greater cortical thickness than those aged ≥60 years in the overall cohort, and this age-related difference appeared more evident in women than in men. These findings are broadly consistent with known age- and sex-related differences in cortical bone morphology and distal fibular microarchitecture [19,22,23]. Although these subgroup analyses were exploratory, they suggest that patient factors such as sex and age may influence the absolute cortical stock available for screw fixation, even when the anterior-to-posterior regional pattern is maintained.

This study has several limitations. First, its retrospective single-center design may limit the generalizability of the findings. In addition, because the study population consisted of patients treated at a single institution in Korea, potential ethnic or population-based differences in distal fibular morphology could not be evaluated and should be considered when applying these findings to other populations. Second, because the cohort included only surgically treated Weber B lateral malleolar fractures with available preoperative CT scans and interfragmentary screw fixation, selection bias may have been introduced, and the cohort may not represent all Weber B lateral malleolar fractures encountered in clinical practice. Therefore, the results may not apply to non-operatively treated fractures, Weber A or C fractures, highly comminuted fractures, or cases treated without interfragmentary screw fixation. Third, BMI was not consistently available in this retrospective dataset; therefore, the potential influence of body mass index or other anthropometric factors on cortical thickness could not be evaluated. Fourth, the measurement method involved matching the interfragmentary screw level on postoperative radiographs to the corresponding level on preoperative CT images. Although this approach was intended to approximate the screw fixation level used during surgery, it cannot perfectly reproduce the three-dimensional screw path. Radiographic projection, limb rotation, screw obliquity, CT reconstruction, slice selection, and fracture displacement may all have influenced the measured values. The measurement procedure was standardized using the lateral malleolar tip as a common reference point and showed good-to-excellent interobserver and intraobserver reliability; however, the validity of radiograph-to-CT matching against the true three-dimensional intraoperative screw path could not be directly quantified. Therefore, the absolute cortical thickness values should be interpreted cautiously, and the results should primarily be understood as demonstrating a consistent relative pattern between the anterior and posterior cortices around the screw fixation level. Fifth, fracture pattern classification was based on diagnostic descriptions, and associated injuries may have introduced heterogeneity into the cohort. Although additional exploratory analyses according to fracture pattern showed consistent anterior cortical predominance across fracture pattern subgroups, this study was not primarily designed to compare fracture pattern groups. Sixth, sex- and age-based subgroup analyses were exploratory and may have been affected by selection factors, injury mechanisms, or fracture characteristics. Finally, this study did not include biomechanical testing or clinical outcome analysis. From a practical perspective, the findings of this study may serve as a basis for future patient-specific fixation planning in Weber B lateral malleolar fractures. Further biomechanical studies are needed to determine whether the observed anterior–posterior cortical difference is associated with screw insertion torque, interfragmentary compression, pull-out strength, or construct stability. Prospective clinical studies could also evaluate whether preoperative assessment of distal fibular cortical morphology influences the choice of screw trajectory, lag screw versus positional screw fixation, or fixation outcomes in patients with poor bone quality. Such studies would help clarify whether the morphometric pattern observed in the present study can be translated into measurable improvements in fixation strategy or clinical results.

In summary, this study demonstrated that the anterior cortex of the distal fibula is consistently thicker than the posterior cortex around the interfragmentary screw fixation level in Weber B lateral malleolar fractures. Although the exact numerical values should be interpreted with caution, this regional morphometric pattern may provide useful reference information when planning screw trajectory, lag screw technique, or positional screw fixation.

## 5. Conclusions

The anterior cortex of the distal fibula was consistently thicker than the posterior cortex around the interfragmentary screw fixation level in surgically treated Weber B lateral malleolar fractures. Given the potential measurement error inherent in radiograph-to-CT matching, the results should be interpreted as demonstrating a relative regional pattern rather than exact absolute cortical thickness values. This morphometric pattern may provide useful reference information when planning screw trajectory and selecting among lag screw fixation, positional screw fixation, or plate-only fixation in individual Weber B lateral malleolar fractures. In selected cases, a posterior-to-anterior lag screw trajectory may be considered so that the relatively thicker anterior cortex functions as the far cortex for screw purchase.

## Figures and Tables

**Table 1:** Patient demographics and fracture characteristics.

Variable	Value
Total patients	155
Age, years	48.1 ± 17.3
**Sex**	
Men	76 (49.0%)
Women	79 (51.0%)
**Injured side**	
Right	72 (46.5%)
Left	83 (53.5%)
**Fracture pattern**	
Isolated lateral malleolar fracture	39 (25.2%)
Bimalleolar fracture or bimalleolar-equivalent injury	39 (25.2%)
Trimalleolar fracture or trimalleolar-equivalent injury	77 (49.7%)

Values are presented as mean ± standard deviation or number (%).

**Table 2:** Comparison of anterior and posterior cortical thickness around the interfragmentary screw fixation level.

Subgroup	n	Anterior Cortex, mm	Posterior Cortex, mm	Mean Difference, mm (95% CI)	*p* Value
Overall	155	2.44 ± 0.82	1.39 ± 0.66	1.05 (0.94–1.17)	<0.001
Men	76	2.66 ± 0.90	1.49 ± 0.76	1.17 (0.99–1.35)	<0.001
Women	79	2.24 ± 0.68	1.30 ± 0.52	0.94 (0.79–1.09)	<0.001
Men < 60 years	55	2.69 ± 0.93	1.54 ± 0.78	1.15 (0.94–1.37)	<0.001
Men ≥ 60 years	21	2.57 ± 0.83	1.36 ± 0.73	1.21 (0.87–1.55)	<0.001
Women < 60 years	47	2.36 ± 0.69	1.39 ± 0.59	0.97 (0.80–1.15)	<0.001
Women ≥ 60 years	32	2.06 ± 0.64	1.17 ± 0.36	0.89 (0.63–1.16)	<0.001

Values are presented as mean ± standard deviation unless otherwise indicated. Mean difference was calculated as anterior cortical thickness minus posterior cortical thickness. CI, confidence interval. *p* values were calculated using paired *t*-tests for within-patient comparisons between anterior and posterior cortical thickness.

**Table 3:** Sex- and age-based subgroup comparisons of cortical thickness.

Comparison	Variable	Group 1	Group 2	*p* Value
Men vs. women	Anterior cortex, mm	2.66 ± 0.90	2.24 ± 0.68	0.001
	Posterior cortex, mm	1.49 ± 0.76	1.30 ± 0.52	0.075
	Mean cortical thickness, mm	2.07 ± 0.74	1.77 ± 0.51	0.004
<60 vs. ≥60 years, overall	Anterior cortex, mm	2.54 ± 0.84	2.26 ± 0.76	0.039
	Posterior cortex, mm	1.47 ± 0.70	1.24 ± 0.54	0.028
	Mean cortical thickness, mm	2.00 ± 0.68	1.75 ± 0.54	0.014
<60 vs. ≥60 years, men	Anterior cortex, mm	2.69 ± 0.93	2.57 ± 0.83	0.581
	Posterior cortex, mm	1.54 ± 0.78	1.36 ± 0.73	0.353
	Mean cortical thickness, mm	2.11 ± 0.76	1.96 ± 0.69	0.412
<60 vs. ≥60 years, women	Anterior cortex, mm	2.36 ± 0.69	2.06 ± 0.64	0.049
	Posterior cortex, mm	1.39 ± 0.59	1.17 ± 0.36	0.044
	Mean cortical thickness, mm	1.87 ± 0.57	1.61 ± 0.37	0.015

Values are presented as mean ± standard deviation. Mean cortical thickness was calculated as the average of anterior and posterior cortical thickness. For the sex comparison, Group 1 indicates men (n = 76) and Group 2 indicates women (n = 79). For the overall age-based comparison, Group 1 indicates patients aged <60 years (n = 102) and Group 2 indicates patients aged ≥60 years (n = 53). For sex-specific age comparisons, Group 1 indicates patients aged <60 years and Group 2 indicates patients aged ≥60 years. *p* values were calculated using Welch’s *t*-test. These subgroup analyses were exploratory.

## Data Availability

The data presented in this study are available from the corresponding author upon reasonable request.

## Abbreviations

The following abbreviations are used in this manuscript:

CT	Computed tomography
CI	Confidence interval
ICC	Intraclass correlation coefficient
IRB	Institutional Review Board
